# Management and organization construction status and development suggestions of aged‐friendly medical institutions in mainland China

**DOI:** 10.1002/agm2.12209

**Published:** 2022-05-30

**Authors:** Hongli Geng, Qiuyun Wang, Jinlong Cui, Qiuyan Gu, Jianjun Long

**Affiliations:** ^1^ Department of Rehabilitation The First Affiliated Hospital of Shenzhen University Shenzhen Guangdong China; ^2^ Yunnan Medical Health College Kunming Yunnan China; ^3^ Xiangya Boai Rehabilitation Hospital Changsha Hunan China; ^4^ Affiliated Maternal and Child Health of Nantong University Nantong Jiangsu China

**Keywords:** age‐friendly, construction status, elderly

## Abstract

The increasing number of regions have begun to construct age‐friendly medical institutions to further promote the “successful aging” of the elderly in mainland China. This study deeply analyzes the development status of age‐friendly medical institutions abroad and describes the policies, research, evaluation, and certification of different countries. This study focuses on the current construction status of age‐friendly medical institutions in mainland China. With the issuing of several national policies, mainland China has established a top‐down system for the construction of age‐friendly medical institutions, which has been gradually implemented in the actions of medical institutions. On the whole, the goal and evaluation standard are clear and the action is rapid. However, it also faces many challenges and problems. This study puts forward various suggestions for the construction of age‐friendly medical institutions, such as increasing manpower and financial investment and carrying out evidence‐based research. In particular, we should pay attention to promoting a bottom‐up construction system, understand the actual needs of the elderly, pay attention to the personal experience of the elderly, and fully mobilize the active and full participation of the whole society.

## Graphical abstract

The increasing number of regions have begun to construct aged‐friendly medical institutions; it brings out many challenges and opportunities. This study puts forward various suggestions for the construction of aged‐friendly medical institutions.

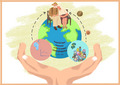

## 1 BACKGROUND

### 1.1 Global aging

The aging population has become a huge challenge facing the world in the 21st century. According to the “World Population Prospects: 2019,”^1^ it shows that by 2050, 16% of people in the world will be at least 65 years old, while the number was 9% in 2019. By 2050, one‐fourth of people in Europe and in North America are 65 years of age or older. In 2018, the global population aged 65 and over surpassed the population under 5 at an unprecedented rate. In addition, the population of 80 years or older is expected to triple, from 143 million in 2019 to 426 million in 2050. Fertility, mortality, and migration are three common factors to contribute to the size and age structure of the population.

Life expectancy^2^ is a key indicator for assessing the health of the population. It has increased significantly in all regions since 1950. The average life expectancy has increased from 45.7 years in 1951 to 72.6 years in 2019 globally. Since 1969, Japan has consistently ranked as the top one country, with 84.6 years life expectancy in 2019.^3^

Children per woman are measured as the total fertility rate.^4^ The global average fertility rate has constantly decreased over the past 50 years and is just below 2.5 children per woman nowadays.

Since immigrants are often young people of working age, they also provide sufficient labor for the destination country. In countries experiencing a large immigration wave, these immigrants will temporarily alleviate the aging trend. However, the immigrants who stay in the country will eventually become the elderly population. Since 1965, the emergence of immigration^5^ has increased the US population from 9.6 million to a record 45 million in 2015. According to the new population forecast of the Pew Research Center, by 2065 there will be 78 million immigrants in the United States. The population growth of the United States is mainly due to the high immigration rate. Although the growth rate has slowed down since 2000, the United States still has the world's largest immigrant population, accounting for about one‐fifth of the world's immigrants. By 2015, the US population will be aging, with an average age of 38. If there were no immigrants, the median age in the country would have been slightly higher since 1965.

### 1.2 Globing coping strategy

As the aging phenomenon intensifies, most of the society domain in the world will be affected, including changes in labor and financial markets, housing, transportation needs, and social security services. Obviously, many countries will face tremendous social, financial, and political pressure. In response to the global issue of aging, the United Nations General Assembly (UNGA) convened an international conference as early as 1982 to address these issues. The conference presented the “The Vienna International Plan of Action on Ageing.”^6^ It includes 62 recommendations, calls for specific actions on issues such as health and nutrition, protection of elderly consumers, housing and the environment, family, social welfare, income security, and employment, education, and research data collection and analysis. In 1991, UNGA claimed the “Plan of Action on Ageing” and designated October 1st each year as “International Day of Older Persons.”

In 2014, the World Health Assembly set the requirements of five key action areas,^7^ which included healthy aging, creating a caring environment for the elderly, adapting the health system to the needs of the elderly population, establishing a system for long‐term care, improving the standard of measurement, monitoring, and research on healthy aging.

Although aging is unavoidable, there are multiple ways to experience aging. Longer life can also be accompanied by staying healthy, participating, and security; then “aging” becomes a positive experience. WHO defined the term “active aging” to this vision. It provides a framework focus on health promotion, participation promotion, strengthening security.^7^

In 2004, WHO^8^ launched the “Age‐friendly Primary Health Care,” aims to improve older adults' care, participation, independence, and dignity. It announced that the information, communication, and training, health care management system, and the physical environment are basic principles during this project. In addition, WHO also provides the “Age‐friendly Tool‐kit,” with training information in testing stages promptly. It will provide the implementation of the Age‐friendly Principles by Primary Health Care (PHC) centers.

In 2019, the National Health Commission has issued the “Guiding Opinions on Establishing and Improving the Elderly Healthcare Service System.” The “Opinions” proposed that “by 2022, more than 80% of general hospitals, rehabilitation hospitals, nursing homes, and primary medical institutions will be friendly medical institutions for elders.”

## 2 DEVELOPMENT STATUS OF AGE‐FRIENDLY MEDICAL INSTITUTIONS ABROAD

### 2.1 The concept of “age‐friendly hospital” was first proposed

An approach to hospital care called age‐friendly hospital was proposed in Canada in 1999.^9^ They found that the elderly make up the majority of all people receiving hospital services in Canada. The current literature is replete with stories of elderly at high risk of hospitalization. Even with the best care after hospitalization, this can lead to longer hospital stays and functional disability. In an atmosphere of austerity, competing priorities, and public pressure, hospitalizing for these people is no easy task. If the number of older persons who received acute care was increased, then the average length of hospitalization would decreased. Acute care hospitals need to rethink how they look after the elderly and also needed a fundamental shift. So they describe the concept of “senior‐friendly hospital” and explain the strategies taken by the Capital Health Region in Canada.

### 2.2 Comparison of the construction of “age‐friendly hospital” in different countries

The research on age‐friendly hospitals is mainly a process of the standards and principles of age‐friendly hospitals established by various scholars in accordance with the national conditions of their own countries. Many countries and regions around the world have carried out unique “geriatric hospital” projects, which have allowed us to achieve various experiences.

In the United States,^10^ the Healthcare Institute and the relevant associations initiated the Age‐Friendly Health Systems, which aims to spread the 4Ms Framework in health care organizations. The 4Ms Framework^11^ stands for the terms “Matters, Medications, Mobility, and Mentation” to address what matters, medications reviewing, keeping elders mobile, and mentation management. Since launching the first AHA Age‐friendly cohort in 2019,^12^ participants in the AHA Age‐Friendly Health Systems Action Community have implemented the 4Ms framework in different care settings. However, a survey^13^ of 479 hospitals found that while 4MS has been proposed and electronic medical records are well established in the United States, there is still a lack of records of elderly information based on the key functions of 4MS. People need to explore how to put 4MS into practical work. The “medical‐orthopedic collaborative work model” reported by Shen et al.^14^ notes that inpatient orthopedic wards can be made into “age‐friendly” fracture patient wards by changing blood draw times, minimizing sleep disruption, and so on to reduce the occurrence of delirium. There are activity experts to provide early activity guidance for these older people.

In Canada, the Canadian Medical Association (CMA)^15^ believes that the governments at all levels should invest in minimizing pressure on the health care system and provide optimal care and support for Canada's aging population. In the CMA recommendations, “high‐quality, well‐funded programs” play a vital role to support physical fitness, optimal nutrition, falls prevention education, and mental health. Its recommendations also complement those made in other CMA policies, including those on “funding continuing care” (2009), optimal prescribing (2010), and drug use and the elderly (2011 update). Mudge et al. ^16^ in Australia opened an investigation to know the perspective on age‐friendly hospitals from different social roles. Finally, the researchers conveyed that the key components of age‐friendly hospitals were that the elderly and their families participate and skilled compassionate staff and care models and environments support older people across the system. During the implementation, empowering local leadership, monitoring, and training are important factors.

Although many countries have promoted “age‐friendly hospitals,” the research on evidence‐based medicine is still bounded; each country and region has formulated its standard rather than the global standard.

The certification of age‐friendly hospitals abroad involves a wide range of targeted standards. The United Kingdom and the Netherlands carried out hospital friendliness certification in 2013 and 2014, respectively, and the certification was carried out in two batches. The Netherlands has developed 15 certification standards, but each one is specific to outpatient wards, and the results of certification are used as a reference for hospital quality improvement. In the UK, accreditation is for individual acute and community wards. Accreditation is divided into two stages: assessing the current quality of care and assessing the quality of continuous improvement.

Based on the experience of other countries, at the national level, corresponding policies and measures should be established to provide medical institutions with a guiding framework for the construction of friendly hospitals, and corresponding evaluation systems and evaluation standards should be put forward. Hospitals should be evaluated regularly using the evaluation standard, and continuous improvement should be made after evaluation. Refer to the WHO concept of “age‐friendly Primary Health Care,” Age‐friendly Primary Health Care Centres Toolkit, and the USA's 4MS framework, and combined with China's actual national conditions, culture, etc., to develop the evaluation system of social, medical institutions in line with China's national needs.

## 3 CURRENT CONSTRUCTION STATUS OF AGE‐FRIENDLY MEDICAL INSTITUTIONS IN MAINLAND CHINA

Mainland China has established a top‐down policy system for the construction of age‐friendly medical institutions, which has been gradually implemented in the actions of medical institutions.

### 3.1 Policies are issued at the national level to clarify the construction contents and requirements

The first guidance document on the elderly health care service system in mainland China was issued on March 30, 2020. This “Opinion” document clarified that by 2022, the systems, standards, and norms related to elderly health care will be basically established, the number of elderly health care service institutions will be significantly increased, the service content will be richer, the service quality will be significantly improved, the service team will be stronger, the service resource allocation will be more reasonable, and a comprehensive and continuous elderly health care service system covering urban and rural areas will be basically established; the health care service needs of the elderly have been basically met. For the first time, the “Opinions” formally proposed to carry out the construction of age‐friendly medical and health institutions and carry out age‐friendly services. Subsequently, the provincial administrative units formulated implementation plans in accordance with the “Opinions.”

In 2020, The (National Health Commission) NHC of the PRC issued the programmatic document “Notice.” It anointed to optimize the medical treatment process for the elderly, provide age‐friendly services, protect the legitimate rights and interests of the elderly, and solve the difficulties encountered by the elderly in intelligent technology. The “Notice” defines the construction content of age‐friendly medical institutions, including the construction of age‐friendly culture, age‐friendly management, age‐friendly services, and an age‐friendly environment.

In 2021, the general office of the NHC issued the “Notice” on the implementation of measures to further facilitate medical treatment for the elderly.”^17^ Ten measurements have been formulated to facilitate the medical treatment of the elderly, including setting up a rapid pre‐examination channel, providing multi‐channel appointment and registration services, optimizing the online and offline service process, providing convenient pharmaceutical services, promoting the “one‐stop” service in and out of the hospital, strengthening the management of hospitalized elderly patients, arranging special personnel to provide medical guidance services, building a medical environment suitable for aging, strengthening the publicity and guidance on the use of intelligent technology for medical treatment of the elderly, and promoting home medical services for the elderly. At the same time, all provinces are required to formulate and implement measures before July 1, 2021 and timely adjust and improve them.

### 3.2 Implementation plans and construction standards were issued at the provincial level, and medical institutions began to implement them

In provinces and municipality, the policies formulated by the State are gradually laying on top down.

Beijing launched the action plan to create an age‐friendly hospital in early 2017, 3 years earlier than the whole country. Beijing Geriatric Hospital, as the geriatric health guidance center in Beijing, is an advocate and pioneer of national age‐friendly hospitals. The hospital first put forward the concept of “creating an age‐friendly hospital” in 2015. The establishment was started in 2016 and an implementation plan was formulated. As of January 2021, 253 medical institutions in Beijing have been rated as age‐friendly medical institutions.^18^ Compared with 2019, Beijing revised the evaluation score in 2020.^19^ It is required that the total evaluation score of the declared medical institution shall not be less than 80 points, and the four aspects evaluation scores of age‐friendly culture, management, service, and environment shall not be less than 80%.

Shanghai aims to create an international age‐friendly city with warm medicine. To this end, more than 70 specific measures have been introduced to improve the rules and regulations and called for the provision of aids for the elderly in the outpatient and emergency departments and the design of conspicuous signs to simplify the inconvenience caused by the use of digital equipment during the epidemic; they only need to swipe the ID card or the social security card can display the health code to optimize the medical process, insist on staff services at the registration window, etc. The first batch of 54 hospitals has been audited and has reached the level of Shanghai's “age‐friendly hospital” standards. Under the guidance of the general policy, different hospitals have also proposed some services with their own features. Since December 2012, Shanghai Huadong Hospital has provided “one‐to‐one” escort services for empty nest, lost, and disabled elderly patients. Jinshan Zhongren Geriatric Nursing Hospital provides barrier‐free travel for elderly patients, and the height of all beds is reduced by 5 cm.^20^

Hunan Province issued the work plan, formulated the construction standard of age‐friendly medical institutions in Hunan Province (for trial implementation), and put forward corresponding work requirements for lower administrative units. Forty‐two days after the release of the work plan in Hunan Province, Changsha County (a county subordinate to Hunan Province) formulated the work plan for the construction of age‐friendly medical institutions in Changsha County and formulated a detailed schedule, construction application form, evaluation and recommendation form, etc. The rehabilitation hospital where the author works is located in Changsha County, which is a class III‐A rehabilitation hospital. According to the field visit, the hospital has actively started the construction of an age‐friendly hospital in accordance with the requirements of the work plan. The hospital has set up a leading group for the construction of an age‐friendly hospital, including team leaders and team members, and defined their work responsibilities. The leading group consists of four working groups headed by the vice president, which are respectively responsible for the creation of management, service, culture, and environment improvement. The task allocation rules for the construction of age‐friendly medical institutions in the hospital were formulated, and the phased promotion scheme and time node were defined. In addition, Hunan province implemented the national policy and issued 10 measures to further facilitate the medical treatment of the elderly on September 18, 2021, which were adjusted on the basis of the measures issued by the State. For example, it is proposed to further improve the medical insurance service management and optimize the remote medical treatment handling services for the elderly.

### 3.3 Research on the construction of age‐friendly medical institutions in mainland China

Although multiple news that the “age‐friendly” policy is being carried out all over mainland China, there are few studies on the construction of age‐friendly medical institutions.

After combing the certification of age‐friendly hospitals, Pan et al.^21^ proposed that China can get the experience of the Netherlands, take combing the concept of age‐friendly service as an important goal of hospital quality improvement, and establish an age‐friendly medical service system. She suggested that hospitals should improve the awareness of humanistic care for elderly patients, strengthen communication with elderly patients, improve hardware settings, pay attention to geriatrics, cultivate a team of professionals, strengthen health education, and encourage elderly patients to actively participate in treatment. Li et al.^22^ explored the establishment of an “age‐friendly” outpatient innovative service model through on‐the‐spot investigation and literature analysis. It mainly includes layered design, characteristic services, and facility transformation. Li proposed that the baseline investigation is the guarantee for carrying out “age‐friendly” services, the application of “quality control circle” management tools is the means to promote “age‐friendly” services, information construction is a double‐edged sword for implementing “age‐friendly” services, hierarchical diagnosis and graded diagnosis and treatment is the breakthrough point for the full implementation of “age‐friendly” services, and the establishment of new doctor–patient relationship is the difficulty and key point to improve “age‐friendly” service; “PDCA” is the highlight and key point.

Li et al.^23^ combined with the characteristics of the elderly, put forward opinions on the construction of barrier‐free facilities in the age‐friendly hospital. Zhao^24^ puts forward the principles of convenience, easy identification, safety, and humanistic care in the design of guidance system for elderly hospitals by analyzing the characteristics and medical confusion of elderly patients, combined with multi‐disciplinary research results, and puts forward specific design methods for relevant elements such as shape, color, font, and position in the guidance system based on theoretical results.

After investigation, Fan et al.^25^ found that PDCA can effectively promote the construction of age‐friendly hospitals. It is mainly reflected in the overall satisfaction of elderly patients and the rescue success rate of elderly critically ill patients. The incidence of adverse events, complications, and mortality decreased compared with those before implementation. Zhang^26^ proposed that the construction of an age‐friendly hospital has promoted the construction of the outpatient system of the elderly hospital.

### 3.4 Problems faced by the construction of age‐friendly medical institutions in mainland China

Zou et al.^27^ compared the research and evaluation status of age‐friendly hospitals in different abroad and analyzed the current situation of age‐friendly hospitals in China. It is proposed that the construction of China's age‐friendly hospital started late, and the main problems are limited research perspective, single research method, insufficient and incomplete development, ignoring the construction of soft environment and cultural level, lack of depth and breadth, etc.

**FIGURE 1:**
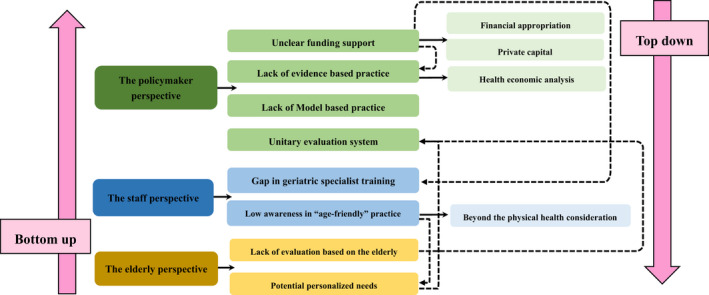
Interaction of existing disadvantage based on various perspectives

Through field visits to medical institutions, the author found that although the construction of age‐friendly medical institutions has been actively responded to and implemented, it still faces many challenges and problems. Although policies on age‐friendly hospitals have been introduced from the country to the region, the financial support for this is equivocal, and there is a lack of a comprehensive training system. From the perspective of the identification standard of age‐friendly hospitals, the evaluation of the subjective feelings of the elderly is ignored. Previous reports focused on the implementation of age‐friendly hospitals but lack of evidence based research on the contribution of age‐friendly hospitals to society (Figure 1).

## 4 SUGGESTIONS

As the existing problems mentioned above, we can classify them into three perspectives, including policymakers, health providers, and the elderly. These three aspects are complementary to each other. Aspects of elder needs provide reflections and ultimately valuable references for policymakers.

### 4.1 Demand and experience of elderly medical patients

To investigate geriatric medical needs in the relevant investigation and research with different age stages, regions, and cultural backgrounds.^28^ These results will reference building age‐friendly hospitals for elderly and improve professions training based on client center approach.

### 4.2 Differentiated and distinctive services

Based on the national requirements of the construction of the standard to differential construction. According to the situation of different hospitals and the needs of local elderly patients,^29^ establish other friendly service systems—effective use of local resources to satisfy the needs of the elderly. Regional resources are effective in establishing a “two‐way” satisfaction medical service system between health providers and the elderly in a regionally differentiated model.^29^, ^30^ The specific content includes dialect services in different regions, food cultures, and economic levels.^31^

### 4.3 Human resource

There are 260 million people who are over the age of 60. There is a severe shortage of medical personnel. The hospital needs to invest more in professional training for geriatric specialists and social workers. Medical universities set up specialized disciplines and train professionals. Take rehabilitation nursing as an example; transfer professionals from existing health care systems for training draw on aboard experience within the situation of mainland China. In addition to increasing clinical expertise, specialists should improve their awareness during clinical practice in psychological and communication skills, change the medical model, and strengthen the friendliness of professionals.^32^

### 4.4 Research on economic and social benefits

There was a limited evidence‐based practice in building age‐friendly hospitals, as far as we know. The government can invest part of the funds in relevant research from which we can detect the cost‐effectiveness of age‐friendly hospitals in terms of health economic benefits, and researchers can also analyze the impact on society from different perspectives. The research results will help policymakers and appropriate professionals find the problems in the construction, adjust strategies, optimize the allocation of resources, and sustainable development.^33^

### 4.5 Financial input

There have been related policies and regulations in relevant government documents to support the construction of age‐friendly hospitals across the country. According to the state's requirements, all provinces and cities are gradually establishing friendly hospitals. Lack of relevant documents to show the source of construction funds for age‐friendly hospitals, so it is suggested that the state increase the investment in the construction of friendly hospitals. On the other hand, the government needs to increase the investment of funds and encourage private capital to invest in the construction of age‐friendly hospitals. This multi‐mode combination effectively promotes the building of an age‐friendly hospital party from the overall medical environment. Financial support can invest in the construction of hardware facilities, such as innovative medical services, professional training, and the improvement of the hardware environment. The government provides financial support and encouragement for the regular evaluation of friendly hospitals based on the elder perspective showing different appeals.

### 4.6 Models based on evidence‐based medicine and continuous improvement

Zou et al.^27^ suggested that we should formulate policies and measures in line with national culture and regional development, formulate investment measures for the construction of age‐friendly hospitals, and pay attention to the transformation of hospital soft services (pre‐hospital communication, reshaping the hospitalization process, extended services), etc.

Mate et al.^11^ called for creating an age‐friendly health system. Becoming an age‐friendly health system entails reliably proving a set of four evidence‐based elements of high‐quality care, known as The 4Ms (an essential set of evidence‐based practices),^17^ to all older adults in your system in an age‐friendly health system. The 4Ms Framework includes What Matters, Medication, Mentation, Mobility.

The key elements of an age‐friendly health system include a plan‐do‐study‐act (PDSA) cycle and setting up a team including certain roles and functions most likely to succeed. Health providers should build the “age‐friendly” care workflow with real‐time observation, improve care, and measure the impact of age‐friendly care. Outcome measures of an age‐friendly health system should include both basic and advanced assessments. The basics include data on admission and discharge, such as 30‐day all‐cause rehospitalization rate, hospital turnover rate, etc.; advanced assessment should concentrate on the elderly need based on mental status indicators.

This change to in the construction process, age‐friendly hospital services refer to relevant research in foreign countries. Continuous adjustments are made in combination with the actual situation, which can help effectively carry out relevant services, effective use of resources, and guidance. Evidence‐based practice can also avoid unnecessary waste of resources and improve the service model’s effectiveness. Grantmakers in aging (2015) describe guiding principles for sustainability beyond provision of funds.

## 5 SUMMARY

In general, this study puts forward two approaches of top‐down and bottom‐up suggestions for China's “Age‐friendly” medical institutions through the analysis and summary of existing research.

However, limited by the lack of existing research, we cannot make more convincing recommendations based on evidence. The absence of such information has also led to a deviation in our understanding of the mainland China situation. Therefore, further studies may require establishing multi‐center collaborative research from the perspective of the elderly, the benefits of hospitals, and social value to negotiate its meaning.

## AUTHOR CONTRIBUTIONS

Each author participated in the writing of this paper, including querying data, arranging and analyzing, and reviewing this article. .

## CONFLICT OF INTEREST

Each author declares that there are no associations of companies that may have a conflict of interest with the submitted article, including but not limited to consulting, equity, equity, patent/license agreements, etc. We have no known conflict of interest to disclose.
